# 7-1-7: the promise of tangible results through agility and accountability

**DOI:** 10.1016/S2214-109X(23)00167-5

**Published:** 2023-04-13

**Authors:** Landry Ndriko Mayigane, Liviu Vedrasco, Stella Chungong

**Affiliations:** a Health Security Preparedness Division, WHO, Geneva 1202, Switzerland

In *The Lancet Global Health*, Aaron F Bochner and colleagues showed how implementing the 7-1-7 target promotes agility and accountability in global health security, using examples from five low-income and middle-income countries.^1^ The Article emphasises the importance of early action to stem morbidity and save lives, as any delays in detection, notification, and response directly affect the outcome of a public health event.^2^ Early action also has applications for animal diseases, including zoonoses, where the impact of a disease, if not quickly contained, could decimate entire populations.^3^

The Article further presents how the timeliness of critical actions matters for effective outbreak management aiming to stop diseases where they occur and as soon as they start. It also provides a quick step-by-step process of review and immediate correction and improvement of the performance of national and local health systems.

The 7-1-7 approach is complementary to the WHO methodologies for intra-action and after-action review^4^ by providing additional agile tools and approaches and supporting the emerging concept of “early action review” for the national and subnational health emergency planners and incident managers to use for rapid performance improvement for outbreak detection and response. It also encourages the principles of gradual learning from experiences and real-world outcomes, reflecting on wins and losses, and adjusting to changing factors as part of continuous improvement. The routine monitoring of detection and response performance through timeliness metrics proposed in this framework is also part of the WHO after-action review guidance published in 2019.^5^

Following WHO's advice, also amplified through a comment published in *The Lancet Global Health* in October, 2020,^6^ many countries adapted and used intra-action reviews as a systems learning and performance assessment tool for course correction and improvement of their national and subnational COVID-19 response strategies. As of March 24, 2023, 145 COVID-19 intra-action reviews have been conducted by 82 countries and reported to WHO since the publication of the guidance for conducting a country COVID-19 intra-action review on July 23, 2020.^7^, ^8^ Countries are already knowledgeable of intra-action and after-action reviews, which is an enabling factor for the 7-1-7 framework to thrive.

According to the WHO global public health intelligence report published in 2022, between 2017 and 2021, the WHO African region reported 589 acute public health events—the highest number among all other WHO regions.^9^ Adoption of the 7-1-7 target for detection, notification, and response to public health threats will contribute to establishing more agile systems especially at local levels that can contain health threats as they emerge on the continent.

To accelerate its uptake, the 7-1-7 target might benefit from the development of a communication package with a clear explanation and justification of why it should take 7 days for detection, 1 day for notification, and 7 days for the response to be completed. Greater clarity around the 7-1-7 target itself would be beneficial. For example, the 7-day target for detection might be challenging to achieve in many less resourced countries with limited access to reliable laboratories for differential diagnosis and where misdiagnosis occurs regularly.^10^ It might similarly be difficult to compute the number of days for detection, especially when there are many probable cases that had not been sampled and confirmed with laboratory testing. Uptake of 7-1-7 could also be accelerated by mainstreaming this target into existing and already established intra-action and after-action review methodologies and regional disease surveillance and response frameworks, such as the Integrated Disease Surveillance and Response in Africa, the Asia Pacific strategy for emerging diseases and public health emergencies for South-East Asia and Western Pacific regions and the Integrated Disease Surveillance in the Eastern Mediterranean region, and the ongoing WHO-led global consultations to strengthen the Global Health Architecture for Emergencies (the Pandemic Accord, the IHR Amendment Process, and the Health Emergency Preparedness, Response and Resilience architecture). Publication of more evidence that shows the impact of countries proactively implementing the 7-1-7 target would aid further uptake and demonstrate its value.

As an Agile framework, 7-1-7 has a lot of value as a complementary approach to intra-action and after-action reviews because it brings additional practical tools to build resilient, flexible, and adaptable health systems, and contributes to strengthening health security preparedness and response in countries. Its further refinement would amplify its impact.

We declare no competing interests. The findings and conclusions in this comment are those of the authors and do not necessarily represent the official position of WHO.
